# Neural regulation of immune evasion in breast cancer: a multiscale neuro–immune framework

**DOI:** 10.3389/fimmu.2026.1859845

**Published:** 2026-07-27

**Authors:** Xingyu Li, Xu Gong, Yizi Cong, Ji Wang, Yansheng Li, Gang Chen, Yalun Li, Guangdong Qiao

**Affiliations:** Department of Breast Surgery, The Affiliated Yantai Yuhuangding Hospital of Qingdao University, Yantai, Shandong, China

**Keywords:** breast cancer, CGRP, immune evasion, metastasis, neuro-immune crosstalk, tumor microenvironment, β-adrenergic signaling

## Abstract

**Background:**

Immune escape remains a major barrier to durable benefit from immunotherapy in breast cancer, particularly in immune-cold tumors and those with an immune-excluded architecture. Emerging evidence from cancer neuroscience suggests that nerves are not passive bystanders of the tumor microenvironment, but active regulators of stromal remodeling, myeloid polarization, T-cell dysfunction, metastatic adaptation, and neuroendocrine stress biology.

**Main body:**

We synthesize current evidence supporting a multiscale model of neuro–immune crosstalk in breast cancer. We first examine sympathetic innervation as an upstream coordinator of immunosuppressive signaling through β-adrenergic pathways that reshape myeloid compartments, lymphangiogenesis, and effector lymphocyte fitness. We then discuss sensory-nerve-driven immune exclusion, focusing on CGRP–CAF–ECM circuits and Substance P-associated inflammatory relays that stabilize prometastatic states. Next, we review direct nerve–tumor interfaces, including neurotransmitter-dependent synapse-like signaling, pseudo-synaptic coupling, extracellular-vesicle/TNT-mediated metabolic communication, and mitochondrial transfer, and evaluate their potential roles in immune resistance and metastatic competence. We further integrate these local interactions into a systems framework by considering tumor–brain–sympathetic feedback loops and neuroendocrine outputs that reset host immune thresholds while emphasizing the context-dependent nature of neural regulation across tumor types and microenvironmental states. Finally, we summarize neurodevelopmental programs co-opted during metastasis, discuss emerging technologies for neural phenotyping and spatial analysis, and highlight clinically actionable vulnerabilities, including β-blockade, CGRP-axis modulation, RET/TRK-targeted therapy, and phenotype-guided combination strategies.

**Conclusion:**

This framework positions neural signaling as an upstream integrator of immune escape in breast cancer and suggests that neural biology may enable biologically informed stratification of immunotherapy-resistant tumors into distinct and targetable states.

## Graphical Abstract

**Figure:**
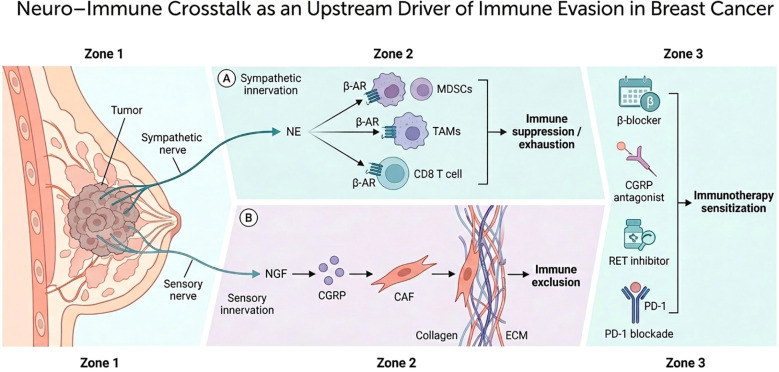
Multi-scale neuro–immune framework for breast cancer immune evasion.

## 1 Background

Breast cancer remains the most frequently diagnosed malignancy in women worldwide, and although immune checkpoint inhibitors have improved outcomes in selected subgroups - especially PD-L1-positive triple-negative breast cancer (TNBC) - the overall response rate remains modest (1–3). A central reason is the persistence of an immunosuppressive tumor microenvironment in which myeloid skewing, T-cell exhaustion, altered vascular and stromal architecture, and metastatic niche adaptation all reinforce immune failure (2, 3). From an immunologic perspective, breast tumors are often categorized according to their spatial immune architecture. “Immune-inflamed” tumors contain functionally active immune-cell infiltration within the tumor core, whereas “immune-excluded” tumors retain immune cells primarily at the stromal margin with limited penetration into tumor nests. By contrast, “immune-desert” tumors exhibit profound paucity of effective immune infiltration altogether. These phenotypes are clinically important because they influence responsiveness to immunotherapy and may arise through distinct microenvironmental mechanisms. Many of the neuro–immune mechanisms discussed in this review are particularly relevant to immune-excluded states, in which stromal remodeling, neural signaling, and spatial barriers collectively restrict effective immune-cell access to tumor tissue.

**Figure 1:**
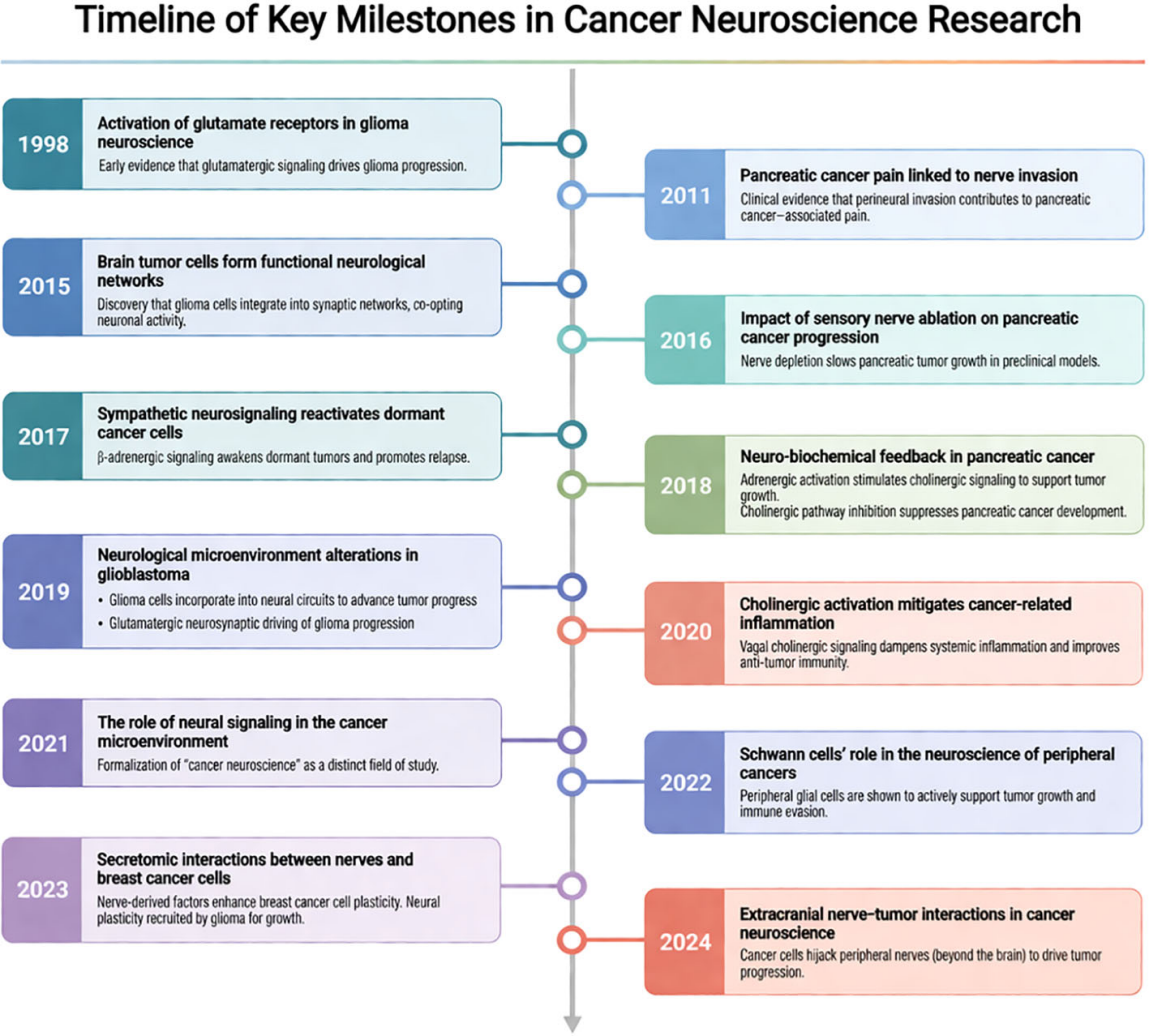
Conceptual evolution of cancer neuroscience and its relevance to breast cancer immune escape.

Historically, nerve involvement in cancer was viewed mainly through the lens of perineural invasion. That view is no longer sufficient. Cancer neuroscience has established that autonomic, sensory, and central neural circuits actively regulate tumor biology, often through pathways that intersect directly with immune control (4–7). This conceptual evolution is summarized in **Figure 1**. In breast cancer, nerve-fiber density is increased relative to normal mammary tissue, with particularly strong signals in aggressive disease and in metastasis-associated contexts (8–10). Clinical and histopathologic observations further support the relevance of neural involvement in breast cancer. In a large retrospective cohort of 8864 patients, perineural invasion (PNI) was identified in approximately 15.6% of invasive breast cancers and was independently associated with increased locoregional recurrence (11). This neural-associated phenotype appears particularly prominent in triple-negative breast cancer (TNBC). In a FUSCC-TNBC cohort (n=465), Zhang et al. further reported that PNI was associated not only with poorer recurrence-free survival, but also with higher nodal stage, increased risk of bone metastasis, and immunosuppressive microenvironmental features (12). Collectively, these findings suggest that neural remodeling in breast cancer is linked to aggressive disease progression and the establishment of an immune-suppressive tumor ecosystem.

The central premise of this review is that nerves act as upstream integrators of immune escape. Rather than serving as one more stromal variable, neural systems influence whether immune cells enter the tumor core, how long they retain effector fitness, whether metastasizing cells can adapt to neural niches, and how systemic stress biology recalibrates host immunity (4, 5, 7, 13–17). This perspective is clinically relevant because it suggests that a proportion of immunotherapy-resistant breast cancers may be neurally organized - and therefore neurally targetable.

An additional conceptual advantage of the neuro–immune perspective is that it links local tumor biology with host state. Classic tumor immunology has long recognized that systemic inflammation, endocrine tone, circadian disruption, and behavioral stress can influence anti-tumor immunity. Cancer neuroscience provides mechanistic pathways through which these host-level variables may become biologically embedded within the tumor microenvironment (14, 15, 18, 19). In this sense, neural signaling does not merely add another ligand-receptor pair to the breast-cancer ecosystem; it provides a route by which central physiology can imprint local immunity, thereby helping to explain why similar genomic tumors may behave differently under different neuroendocrine conditions.

To address these challenges, this review synthesizes current evidence on neural regulation of immune evasion in breast cancer at multiple levels. It first consolidates the strongest data supporting the view that nerves act as upstream determinants of immune dysfunction rather than passive correlates of aggressive disease. It then distinguishes mechanisms supported by breast-cancer-specific evidence from those primarily inferred from other solid tumors but likely to be relevant. Finally, it outlines a translational framework in which neural features may stratify breast cancers into intervention-relevant phenotypes, including adrenergic-dominant, sensory-exclusion-dominant, interface-dominant, and systems-neuroendocrine-dominant states (5–7, 16). Such stratification is particularly relevant because it links microenvironmental immunobiology to therapeutic design, biomarker development, and prospective trial architecture.

A second issue is heterogeneity. Breast cancer is not uniformly innervated, uniformly inflamed, or uniformly stress-responsive. Triple-negative tumors often show the clearest linkage between neural remodeling, inflammatory rewiring, and immunotherapy sensitivity, but luminal tumors can also exploit neural programs during endocrine resistance, dormancy, and metastatic colonization (8–10, 14, 16, 20). Likewise, the immune consequences of neural input are likely to differ among immune-inflamed, immune-excluded, and immune-desert states. A neurally amplified immune-desert tumor may be dominated by impaired antigen presentation and myeloid suppression, whereas a neurally amplified immune-excluded tumor may be dominated by matrix architecture, vessel dysfunction, and peritumoral immune trapping. Recognizing this distinction is important for both experimental design and therapeutic prioritization.

**Figure 2:**
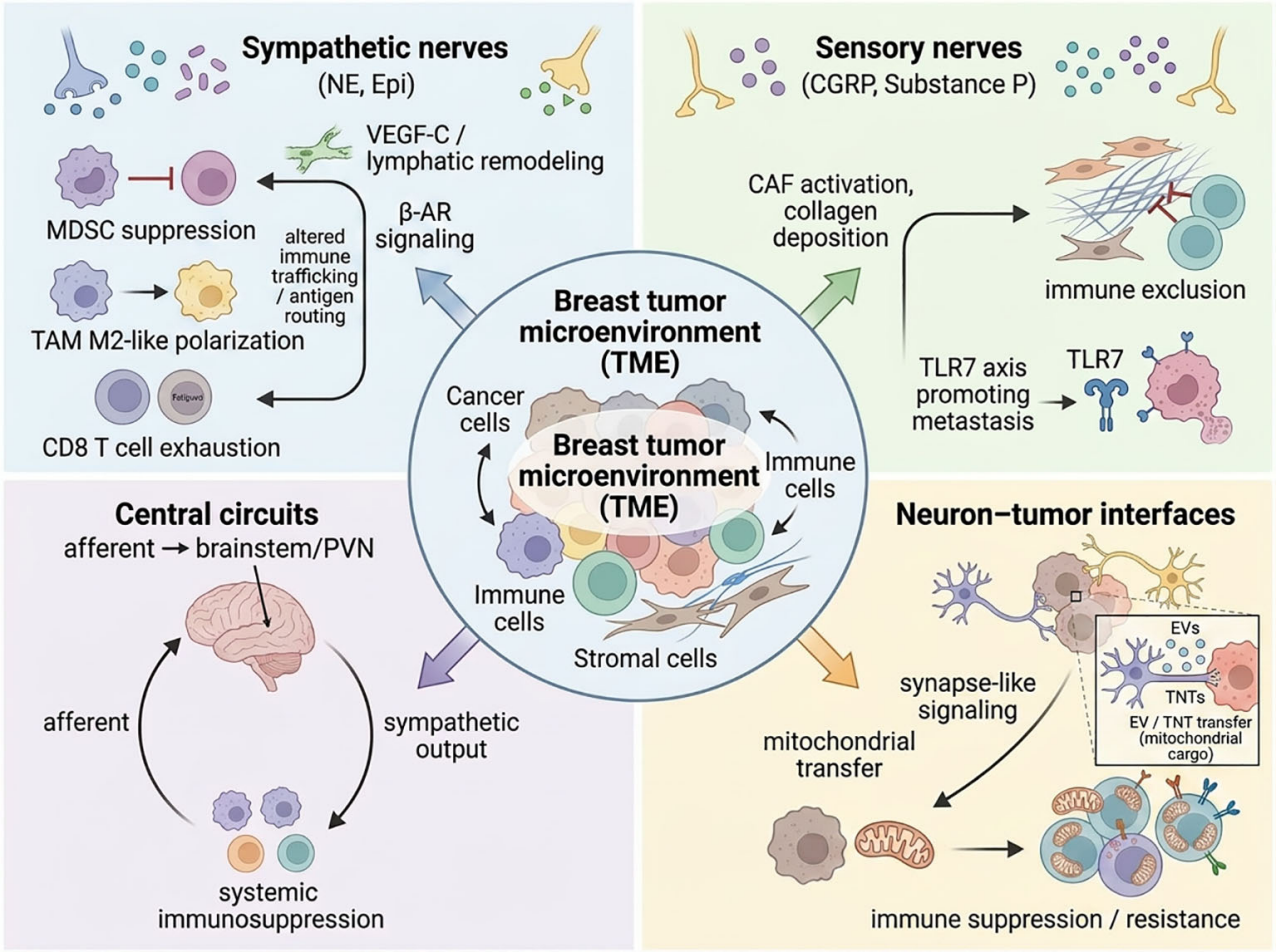
Mechanism overview: four layers of neural regulation of antitumor immunity in the breast tumor microenvironment.

**Figure 3:**
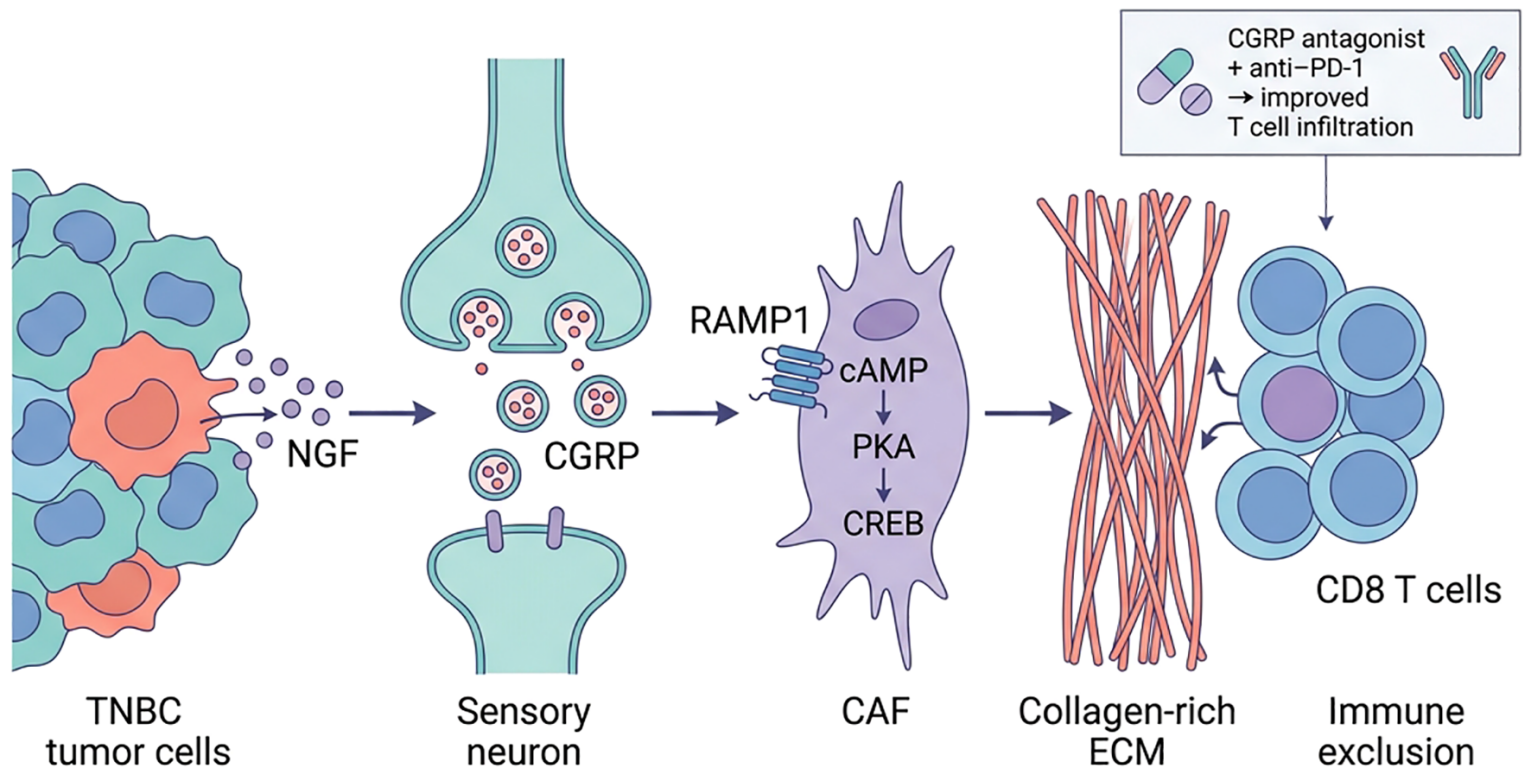
Pathway detail: sensory neuron NGF→CGRP→CAF (RAMP1)→cAMP/PKA/CREB→collagen-rich ECM drives immune exclusion in TNBC and can be therapeutically targeted.

**Figure 4:**
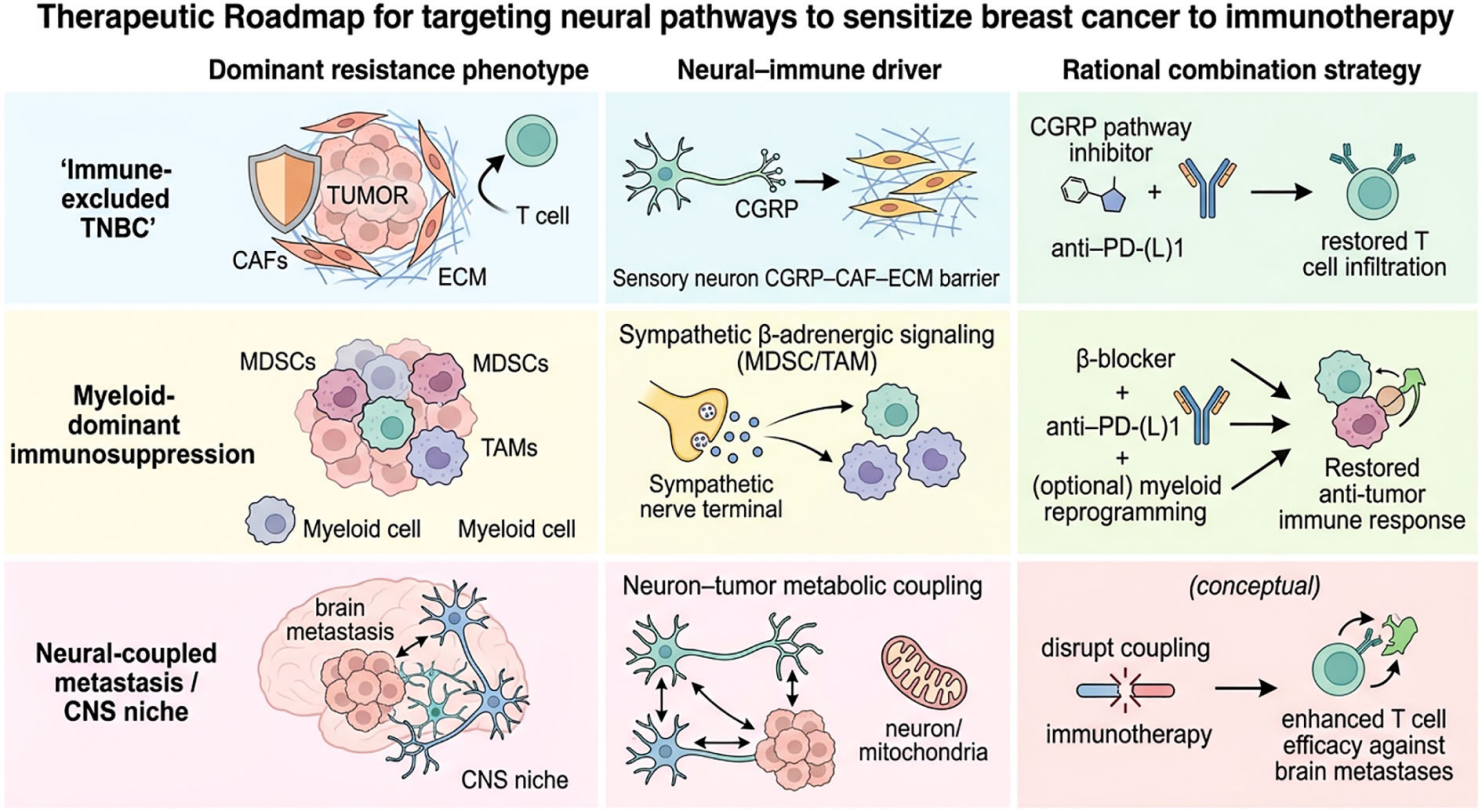
Therapeutic roadmap: rational combinations that target neural pathways to sensitize breast cancer to immunotherapy.

A complementary issue is the immunologic definition of immune escape. In breast cancer, immune escape is not a single event but a composite phenotype that includes inadequate antigen presentation, exclusion of effector cells from the tumor core, myeloid dominance, impaired dendritic cell priming, dysfunctional interferon signaling, and the establishment of metastatic niches that suppress local cytotoxic immunity before clinically detectable dissemination occurs (2, 3). This matters because neural regulation can intersect with each of these layers at different spatial scales. Adrenergic signaling primarily alters leukocyte state and trafficking; nociceptor-derived signals can harden stromal barriers and reshape vascular access; direct neuron-cancer interfaces may increase metabolic fitness and resistance to death; and central neuroendocrine outputs can reset systemic inflammatory thresholds. The neuro–immune model is therefore not a competing explanation for immune escape, but an organizing framework that connects several otherwise fragmented observations into a hierarchical system (**Figures 1**–**4**). This review focuses on breast-cancer–validated mechanisms, while clearly distinguishing extrapolated concepts.

## 2 Neural architecture of immune escape in breast cancer

Three partially overlapping levels of neural regulation appear especially relevant in breast cancer. The first is local autonomic regulation, dominated by norepinephrine-driven adrenergic signaling. The second is sensory-nerve regulation, which is increasingly implicated in immune exclusion, matrix remodeling, and inflammatory amplification. The third is systems-level integration, in which tumor-derived signals access central circuits and neuroendocrine outputs (6, 10, 14–16, 21–23).

These levels are not equivalent. Sympathetic signaling tends to act most strongly on immune-cell function and vascular or lymphatic remodeling, whereas sensory signaling can reshape the physical and spatial logic of immune-cell entry. In parallel, direct neuron-cancer interfaces and metastatic neural adaptation can support therapy resistance even when classical immune checkpoints are targeted (9, 21–26). These interacting layers are summarized in **Figure 2**.

Another practical distinction is between acute neural signaling and chronic neural state. Brief bursts of sympathetic activity may transiently alter trafficking or vascular permeability, whereas chronic stress-associated adrenergic exposure can progressively reprogram tumor and immune compartments into a stable prometastatic configuration. Similarly, transient nociceptor activation may accompany tissue injury, but persistent sensory remodeling can create a long-lived matrix-rich, exclusionary niche. The breast-cancer field increasingly needs longitudinal rather than purely cross-sectional models to capture these dynamics, particularly when integrating surgery, pain, perioperative stress, neoadjuvant therapy, and evolving immune contexture into a single biologic narrative (12, 14, 18, 19, 23).

For immunologists, one useful way to conceptualize these layers is to distinguish between functional suppression and spatial suppression. Functional suppression refers to states in which cytotoxic lymphocytes or antigen-presenting cells are present but biochemically restrained. Spatial suppression refers to states in which immune cells fail to reach relevant tumor compartments, remain sequestered in stromal zones, or are diverted into lymphatic or perivascular circuits that do not support tumor killing. Neural signals are unusually well positioned to control both dimensions because nerves directly regulate vascular tone, stromal-cell activation, matrix deposition, cytokine gradients, and immune-cell receptor signaling. This dual control may explain why neural crosstalk can generate immune phenotypes that are more durable than those created by any single cytokine pathway (12, 14, 16, 23, 27–29).

### 2.1 Sympathetic innervation and β-adrenergic–mediated immunosuppression

The sympathetic axis is the best-established neural pathway in breast cancer progression. Tumor cells and stromal compartments respond to norepinephrine and epinephrine through β-adrenergic receptors, especially β2-AR, and chronic stress can amplify this signaling over prolonged periods (14, 15, 24–26, 30, 31). ^A^ useful conceptual distinction is between the effect of sympathetic signaling on tumor cells themselves and its effect on the immune ecosystem. The latter is more relevant for immune escape.

At the tumor-cell level, β-adrenergic signaling can promote invasion, invadopodia formation, epithelial-mesenchymal plasticity, angiogenesis, and lymphangiogenesis (21–23, 27–29, 32, 33). In breast-cancer models, chronic stress increases VEGF-C output and remodels the lymphatic network, thereby facilitating dissemination while also altering routes of immune-cell trafficking (12, 23). Lymphatic vessels actively regulate the transport of tumor antigens and the migration of dendritic cells (DCs) from peripheral tissues to draining lymph nodes, where effective T-cell priming is initiated. Excessive or aberrantly organized lymphangiogenesis may disrupt this process by redirecting antigen flow, altering DC maturation and trafficking dynamics, and promoting tolerogenic signaling within lymphatic niches (34, 35). As a consequence, antigen presentation may become less effective at generating productive cytotoxic T-cell responses, thereby weakening immune surveillance despite ongoing immune-cell infiltration. This is an important point: neural regulation does not merely suppress immune-cell function; it can also change the structural geography in which immune surveillance occurs.

At the myeloid level, β-adrenergic signaling enhances the immunosuppressive program of myeloid-derived suppressor cells (MDSCs), partly through STAT3-associated pathways and the upregulation of suppressive mediators such as Arg1 and PD-L1 (24, 25, 36, 37). The quantitative accumulation of MDSCs is not always the decisive feature; in several settings the dominant effect is functional reprogramming. Tumor-associated macrophages (TAMs) are similarly affected. Stress-associated catecholamine exposure increases M2-like polarization and the expression of IL-10, Arg1, and chemokines such as CCL22, thereby strengthening a tolerant microenvironment (26, 30, 38).

Adaptive immunity is also directly vulnerable to adrenergic control. β-adrenergic signaling can reduce IFN-γ production, increase PD-1 expression, weaken memory differentiation, and bias T cells toward a hyporesponsive state (30–32, 39). Additional systemic endocrine outputs - especially glucocorticoids - compound this effect by suppressing antigen presentation, dampening NK-cell activity, and restraining cytotoxic responses (40, 41). Collectively, the adrenergic axis behaves as an upstream coordinator of immune suppression rather than as an isolated tumor-cell growth pathway.

Taken together, sympathetic signaling can be understood as a multicomponent immunologic governor. It increases the supply and suppressive potency of myeloid cells, lowers effector T-cell fitness, weakens innate surveillance, and remodels lymphovascular architecture in ways that facilitate both dissemination and immune avoidance (14, 15, 21–26, 30–32). The strength of evidence for each individual node varies, but the network-level direction of effect is remarkably consistent across experimental systems.

Natural killer cells add another important dimension. NK cells contribute to early control of disseminated tumor cells, yet their activity is highly sensitive to neuroendocrine regulation, including glucocorticoids and catecholamines (32). In breast cancer, where metastatic relapse often reflects failure to eliminate residual or disseminated disease, any neural program that weakens NK surveillance could have consequences that are temporally decoupled from the primary tumor. This possibility reinforces the idea that sympathetic biology may matter most not only for primary tumor growth but also for occult dissemination and niche conditioning.

The adaptive compartment should also be viewed through a temporal lens. Early adrenergic signaling may transiently alter T-cell migration or cytokine secretion, but chronic signaling appears more likely to consolidate exhaustion-associated states, including reduced IFN-gamma production, impaired cytotoxicity, and increased inhibitory receptor expression (30, 31). This distinction may be clinically relevant in perioperative and neoadjuvant settings. Interventions timed to windows of maximal neural stress might yield disproportionately large immunologic benefit even if the same intervention has weaker effects when delivered later in the disease course.

Adrenergic control also intersects with antigen presentation. Although direct breast-cancer data remain less extensive than data for myeloid polarization, several lines of evidence suggest that chronic catecholamine exposure can weaken dendritic-cell maturation, limit effective priming, and reduce the quality of type I or type II interferon-associated anti-tumor responses (14, 15, 30). The likely consequence is a layered immunologic defect: fewer competent dendritic cells, more suppressive myeloid cells, and exhausted or metabolically compromised effector lymphocytes. Such a configuration would be expected to reduce the probability of durable response to checkpoint blockade, even in tumors with measurable PD-L1 expression.

The myeloid compartment appears particularly sensitive to adrenergic tone. Beyond enhancing canonical MDSC suppressive programs, β-adrenergic signaling can reinforce macrophage polarization toward tissue-remodeling, angiogenic, and T-cell-suppressive phenotypes while simultaneously shaping cytokine and metabolite outputs that propagate immunosuppression to neighboring cell populations (24–26). In this setting, macrophages are not just responders to tumor-derived cytokines; they become relay nodes that translate neural information into microenvironmental architecture. This relay function may be especially important in breast cancer, where TAM abundance often correlates with poor outcome, therapy resistance, and metastatic competence.

At the vascular-stromal interface, β-adrenergic signaling can amplify immune escape by changing how tumor territory is physically organized. Catecholamine exposure can increase angiogenic and lymphangiogenic programs, alter endothelial permissiveness, and support fibroblast states that favor matrix deposition and chemokine gradients incompatible with deep cytotoxic T-cell penetration (12, 21–23, 26). These effects are immunologically consequential because the quality of anti-tumor immunity depends not only on whether leukocytes are activated, but also on whether they can enter, persist within, and navigate the tumor core. Neural control of vessel behavior and lymphatic remodeling therefore deserves to be considered part of immune escape biology, not merely a metastasis accessory.

One underappreciated feature of sympathetic regulation is the existence of feedforward trophic loops. Tumor cells and activated stromal cells can produce neurotrophic cues such as NGF and BDNF that attract or stabilize adrenergic fibers, while adrenergic signaling in turn promotes additional stromal activation, inflammatory mediator release, and tissue remodeling that favor further neural persistence (9, 10, 17, 20, 42). Breast-cancer-specific evidence further supports this feedforward concept: in TNBC, norepinephrine/β2-adrenergic signaling has been shown to promote cancer-cell proliferation and increase NGF production, suggesting that sympathetic input may reinforce tumor innervation through tumor-cell-derived neurotrophic output (43). This means that neural abundance in the tumor bed may be both a consequence and a driver of aggressive biology. In practical terms, studies that treat nerve density only as a biomarker risk missing the causal reciprocity of the system.

At the systems level, psychosocial and behavioral states may further influence sympathetic and neuroendocrine signaling relevant to tumor immunity. Chronic stress, sleep disruption, social isolation, and physical inactivity have all been associated with altered catecholamine release, glucocorticoid signaling, and inflammatory regulation, potentially shaping the breast-cancer immune microenvironment through host-level neuro–immune mechanisms. Consistent with this view, clinical studies in breast-cancer patients have linked depressive symptoms and psychosocial distress to elevated TNF-α, IL-1β, and IL-6 levels, increased expression of pro-inflammatory and tissue-remodeling genes, and poorer long-term survival outcomes (18, 44–48). Although the mechanistic basis and clinical magnitude of these effects remain incompletely defined, emerging evidence suggests that behavioral interventions—including exercise, stress-management strategies, and mindfulness-based approaches—may partially modulate systemic inflammation and neuroendocrine activation. These observations support the broader concept that neural regulation of tumor immunity extends beyond the tumor microenvironment itself and may also involve organism-level physiologic states (49).

### 2.2 Sensory nerves, matrix remodeling, and immune exclusion

In contrast to the sympathetic axis, the sensory axis appears especially important for spatial immune failure. The concept of immune exclusion is useful here: effector lymphocytes may be present at the invasive margin but remain unable to enter the tumor core. Recent breast-cancer-specific work now links this phenotype to sensory neurons, CGRP signaling, and matrix remodeling (8, 9, 29, 50–53).

A mechanistic model has emerged in which tumor-derived NGF and inflammatory mediators promote nociceptor sprouting and activation. Activated sensory fibers release CGRP, which acts on stromal compartments - particularly CAFs - to trigger a cAMP-PKA-CREB program that increases collagen deposition and matrix density (29, 50, 51, 54). The immunologic consequence is not only stromal stiffness; it is a change in the access architecture of the tumor. In practical terms, the matrix becomes a neuralized barrier. This sensory neuron–CAF–ECM pathway is illustrated in **Figure 3**. A recent breast-cancer-specific study provides strong evidence for this mechanism, although validation across independent models remains limited.

Sensory signaling is also not limited to CGRP. Substance P signaling has been shown to drive extracellular RNA–TLR7 pathways and pro-metastatic IL-1β-positive neutrophil programs, including in breast cancer models, although some mechanistic insights derive from other tumor systems (36, 37, 55). In this context, neutrophil extracellular traps (NETs) represent a key downstream effector mechanism, as they have been shown to promote metastatic seeding, awaken dormant tumor cells, and contribute to an immunosuppressive microenvironment in breast cancer (56). Thus, sensory nerves may convert local tissue injury and neurogenic inflammation into a prometastatic, immune-excluded state.

This framework also helps reconcile apparently contradictory observations. Sensory fibers can be anti-tumor in some experimental contexts, especially when acute nociceptive activation strengthens T-cell recruitment. However, once the stromal response becomes chronic and matrix-dominant, the net effect can switch toward stable immune exclusion (19, 29, 51). The balance between acute immune stimulation and chronic stromal locking likely represents a critical but still poorly resolved variable.

From a translational perspective, sensory-exclusion biology may help explain why some checkpoint-refractory tumors do not respond even when there is evidence of antigenicity or peripheral immune activation. If T cells cannot traverse a neurogenically remodeled stromal corridor, or if NK-cell recruitment is suppressed while vessels and matrix are reorganized into exclusionary geometries, systemic immune activation alone may be insufficient. In such patients, the logical intervention may be to first soften the barrier state by targeting the neural circuit that sustains it, then introduce or intensify immunotherapy.

Importantly, sensory nerves are unlikely to exert a uniform effect across all breast-cancer settings. In some models, neuronal activity may transiently promote local inflammatory recruitment or interact with tissue repair in ways that are not straightforwardly pro-tumor. The dominant effect probably depends on duration, fiber subtype, mediator repertoire, tissue context, and the balance between anti-tumor immune stimulation and barrier-forming stromal activation. Careful temporal and spatial phenotyping is therefore essential before generalizing across tumor subtypes or therapeutic settings (12, 27–29).

Another reason to emphasize sensory biology is that pain-related phenotypes may eventually provide clinically accessible surrogates of tumor-nerve interaction. Although pain intensity is nonspecific and influenced by many variables, persistent nociceptive activation, sensory fiber sprouting, or neuropeptide signatures in tumor-adjacent tissue could capture aspects of disease biology that are invisible to standard pathology. This idea remains preliminary in breast cancer, but it is attractive because it links symptom biology with immune architecture and could help identify patients in whom sensory-targeted strategies are most relevant (8, 12, 27, 28, 32).

Substance P-associated pathways extend this model from structural exclusion to inflammatory amplification. Rather than producing a classically anti-tumor inflammatory milieu, nociceptor-driven Substance P signaling can favor inflammatory states that are simultaneously tissue-remodeling and immunosuppressive, for example through extracellular RNA-TLR7 signaling, recruitment of neutrophil subsets with tumor-promoting features, and cytokine circuits that facilitate metastatic seeding (36, 37). This is an important conceptual shift: pro-inflammatory does not necessarily mean immunologically competent. In sensory-driven tumors, inflammation may coexist with poor tumor control because the inflammatory program is organized around wound-like repair, matrix turnover, and metastatic permissiveness rather than antigen-specific clearance.

The CGRP axis is particularly instructive because it connects neuronal activity to fibroblast behavior, extracellular matrix density, vascular remodeling, and lymphocyte function within a single circuit. Breast-cancer-specific work suggests that sensory neurons can stimulate CAFs to deposit and organize matrix in a way that physically constrains immune-cell access, while CGRP may also blunt cytotoxic programs in CD8-positive T cells and NK cells (12, 27, 29). These effects are not merely additive. Dense ECM promotes an immune-excluded architecture characterized by impaired CD8 T-cell penetration. A dense, misaligned matrix increases the distance between effector cells and malignant cells, and when combined with partial lymphocyte dysfunction, even modest spatial barriers may become biologically decisive.

Sensory regulation deserves equal attention because it may help explain a major paradox of breast-cancer immunobiology: some tumors contain immune cells at the invasive margin yet remain poorly penetrated and clinically resistant. In such cases, immune failure is not adequately captured by bulk measures of infiltration. Rather, the problem is topographic. Sensory neurons are increasingly implicated in creating a stromal and vascular geography that channels immune cells into peripheral or dysfunctional positions while preserving a protected tumor core (12, 27–29).

### 2.3 Direct neuron-cancer interfaces and metabolic coupling

An additional level of regulation is emerging from studies of direct neuron-cancer connectivity.

#### 2.3.1 Synapse-like signaling and neurotransmitter coupling

The clearest evidence for neuron-to-tumor synaptic communication has emerged from glioma and small-cell lung cancer, where functional synapse-like structures permit highly localized neuronal signaling. In pancreatic cancer, sensory-neuron-cancer pseudo-synapses based on glutamatergic signaling have also been demonstrated convincingly. Comparable structures have not yet been definitively established in breast cancer. Nevertheless, breast-cancer cells express multiple neurotransmitter receptors and exhibit strong evidence of neural adaptation, particularly in neural-rich metastatic niches (18, 20, 38, 41, 57, 58).

One breast-cancer-relevant example is GABA biology in brain metastasis. Breast-cancer cells in the neural niche can take up and metabolize GABA, using it not only as a neurotransmitter-associated signal but also as a metabolic substrate, thereby enhancing metabolic flexibility (39, 41). This adaptation may help support survival in the brain, where the immune context is specialized and distinct from that of extracranial disease. The immune consequence is indirect but important: metabolically adapted tumor cells are often better able to compete for nutrients, tolerate oxidative stress, and survive under therapeutic pressure.

Pseudo-synaptic signaling provides a related but conceptually distinct mechanism. In pancreatic cancer, sensory-neuron-cancer pseudo-synapses based on glutamatergic signaling have now been demonstrated convincingly (59). Comparable structures have not yet been definitively proven in breast cancer, but breast-cancer cells express multiple neurotransmitter receptors and already show strong evidence of nerve-guided migration and neural adaptation (9, 39, 58–60). These observations suggest that neural circuits may regulate tumor behavior with greater spatial and temporal precision than previously appreciated.

#### 2.3.2 Extracellular vesicle/TNT-mediated metabolic coupling and immune suppression

Beyond neurotransmitter-dependent signaling, neuron–tumor interactions may also involve the direct transfer of bioactive cellular cargo. Tunneling nanotubes (TNTs) and organelle transfer create a metabolic bridge between nerves and cancer cells. TNTs are thin, F-actin–based membranous protrusions that establish direct cytoplasmic continuity between cells, enabling the intercellular transfer of organelles, proteins, and signaling molecules (61, 62). In parallel, extracellular vesicles (EVs) can mediate longer-range transfer of metabolic, neurotrophic, and immunomodulatory cargo, together forming a broader system of intercellular metabolic coupling (63, 64).

Recent work has shown that mitochondria can move from nerves into cancer cells, increasing oxidative phosphorylation and enhancing metastatic fitness (41). Although this phenomenon has not yet been fully mapped in breast cancer, accumulating evidence indicates that intercellular mitochondrial transfer is a biologically relevant process in the breast tumor microenvironment. For example, stromal cells such as cancer-associated fibroblasts can donate mitochondria to tumor cells, enhancing migratory capacity and supporting aggressive phenotypes (65). In addition, breast-cancer-specific studies demonstrate that mitochondrial transfer can occur between mammary epithelial cells and cancer cells primarily through TNTs, leading to metabolic reprogramming and redirection of tumor cell fate (66). Recent evidence further suggests that EV/TNT-mediated transfer may contribute not only to metabolic adaptation but also to immune suppression. TNT-mediated mitochondrial transfer has been shown to regulate macrophage metabolic fitness and inflammatory polarization, supporting the broader concept that organelle sharing can reshape immune-cell function within diseased microenvironments (67). By enhancing oxidative phosphorylation, redox buffering, and resistance to inflammatory stress, transferred mitochondria may reduce immunogenic stress signaling and support tumor persistence under immune pressure. Tumor-derived EVs have been shown to remodel immune-cell function by altering macrophage polarization, impairing dendritic-cell activity, and metabolically reprogramming T-cell responses, thereby contributing to immune suppression within the tumor microenvironment (68). EV-associated cargo—including metabolites, mitochondrial components, cytokines, microRNAs, and neurotrophic factors—may further reinforce stromal activation, inflammatory adaptation, and niche permissiveness across spatially separated cellular compartments (69–71). Recent breast-cancer-specific evidence further suggests that extracellular vesicles may facilitate brain-metastatic adaptation by remodeling blood–brain barrier endothelial-cell dynamics and promoting transcytosis-associated niche permissiveness, thereby supporting early metastatic colonization within neural-rich microenvironments (72).

More broadly, intercellular networking within the tumor microenvironment—including interactions with tumor-associated macrophages—may also involve TNT-mediated communication, highlighting mitochondrial transfer as a generalizable mechanism of microenvironmental regulation (73). Taken together, these findings suggest that neuron-derived mitochondrial transfer represents one component of a broader metabolic coupling paradigm, in which diverse cellular sources within the tumor microenvironment contribute to tumor fitness. In this context, neuron-supported metabolic resilience may be particularly relevant to immune resistance and the escape from metastatic dormancy in breast cancer (74).

In practical terms, interface-dominant tumors may require distinct therapeutic logic. Such tumors may not be adequately identified by PD-L1 expression, tumor-infiltrating lymphocyte (TIL) counts, or global neural-gene signatures alone, but instead may require composite biomarkers integrating neural-contact structures, neurotransmitter receptor localization, metabolic adaptation, and niche-specific dependencies. This may be particularly relevant in breast-cancer metastasis to the brain and meninges, which have become informative settings for studying cancer neuroscience in an immunologic context (39–41, 51, 57).

Several recent high-resolution studies have provided increasingly direct evidence that neurons can establish highly organized synapse-like interfaces with tumor cells (75, 76). In glioma and other neurotropic malignancies, these interfaces permit spatially restricted neurotransmitter signaling, electrophysiologic coupling, and sustained neuronal input that can reshape tumor-cell metabolism, stress adaptation, and invasive behavior. Importantly, such interactions are not merely structural contacts but functionally active communication platforms capable of supporting tumor fitness within neuralized microenvironments (77). Although the clearest evidence currently derives from glioma and other non-breast tumor models, these findings have important conceptual implications for breast cancer, particularly in neural-rich metastatic niches such as the brain and meninges (78). In these settings, localized neuron–tumor interfaces may support metabolic adaptation, stress tolerance, and persistence under immune pressure, thereby contributing indirectly to local immune suppression and treatment resistance.

Pseudo-synaptic and glutamatergic mechanisms may also matter even when definitive breast-cancer-specific evidence is incomplete. If breast-cancer cells use receptor-enriched membrane domains to capture directional neuronal input, then neural circuits may shape invasive behavior and treatment resistance with a precision that bulk transcriptomics cannot easily infer (40). This is particularly relevant for heterogeneous tumors in which only a subpopulation may become interface-competent. Spatially resolved approaches and functional co-culture systems will be required to detect these rare but potentially high-impact states.

Breast-cancer cells within the neural niche can utilize GABA to support anabolic metabolism and redox balance, thereby enhancing survival in an otherwise hostile microenvironment (39, 41). Such metabolic flexibility may indirectly promote immune evasion by reducing immunogenic stress and supporting persistence under inflammatory pressure (79). Although the immune landscape of brain metastasis differs from that of the primary tumor, the principle that neural metabolites can be repurposed into immune-relevant fitness advantages likely generalizes.

Direct neuron-cancer interfaces are conceptually important because they move the field beyond the idea that nerves act only through diffuse neurotransmitter clouds. Structural coupling allows tumors to access high-fidelity, localized, and sustained neural information, potentially conferring advantages that are difficult to interrupt with standard immunotherapies (38–41, 51). From an immunology standpoint, the key question is not merely whether such interfaces increase proliferation, but how they alter the metabolism, stress tolerance, and secretory phenotype of cancer cells in ways that feedback on immune surveillance.

### 2.4 Central circuits, neuroendocrine outputs, and systemic immune thresholds

Local neural regulation is only part of the story. Tumors can also engage the central nervous system through sensory afferents and humoral signals. Recent studies indicate that tumor-derived mediators such as LIF and Gal3 activate hypothalamic stress circuits, increase sympathetic output, and restrain antitumor immunity through a sensory-sympathetic axis (51, 53, 54, 80–83).

This circuit-level perspective is especially important for interpreting chronic stress in breast cancer. Stress is not simply a psychological co-variable; it is a biologic amplifier that can convert local inflammatory or nociceptive signals into systemic immune recalibration (15, 19, 31, 41, 81). In breast-cancer models, functional brain-tumor circuits have been reported, and central modulation of these circuits alters tumor progression (58, 80).

The CNS therefore acts as a state-setting regulator. Through combined sympathetic and HPA-axis outputs, it lowers the threshold for immune exhaustion, weakens innate immune surveillance, and sustains a host environment permissive for progression (18, 19, 80, 82, 83). Conceptually, this means that some breast tumors may remain locally targetable yet systemically protected by neural state regulation. That insight argues for combining local immune interventions with strategies that interrupt circuit-level amplification.

The major translational implication of this framework is that tumor immunity should, in certain contexts, be modeled as a brain–body–tumor network rather than a tumor-confined process. This perspective does not diminish the value of genomic or local microenvironmental analyses; instead, it highlights that these layers may be incomplete when interpreted in isolation. Integrative approaches that incorporate behavioral, endocrine, autonomic, and spatial tumor parameters are therefore more likely to yield clinically relevant mechanistic insights than single-compartment analyses alone.

The systems view also encourages a broader interpretation of immune escape. A tumor does not need to silence every immune effector mechanism locally if the host environment is repeatedly reset toward tolerance, myeloid mobilization, and lymphocyte dysfunction. This may be especially relevant for minimal residual disease and metastatic dormancy, where small shifts in systemic immune threshold could determine whether disseminated cells are held in check or permitted to expand. Neural-state interventions may therefore be most effective in adjuvant or early metastatic settings, even though they are often first tested in advanced disease.

A related issue is reciprocity. Tumors can generate pain, inflammatory mediators, and damage-associated cues that activate sensory pathways projecting to the CNS, while the CNS can respond by increasing sympathetic outflow or glucocorticoid exposure, producing a self-reinforcing circuit (50, 52, 53, 55, 58). Recent work further suggests that neuro–immune regulation may involve anatomically specialized immune trafficking routes. For example, calvarial bone marrow–derived immune cells can be mobilized and recruited into the central nervous system through skull–meningeal connections, highlighting a previously underappreciated axis linking neural niches and immune cell dynamics (84). Such reciprocity helps explain why tumor progression and stress phenotypes can become mutually amplifying rather than merely correlated. It also suggests that supportive care domains often treated as parallel to anticancer therapy may, in some patients, intersect directly with tumor immunobiology.

Breast cancer is particularly suitable for this perspective because disease course often includes prolonged prediagnostic stress exposure, perioperative neuroendocrine perturbation, repeated systemic therapy, and metastatic transitions into organs with strong neural regulation. In this context, the CNS does not simply modulate mood or behavior; it functions as a physiologic integrator that can alter monocyte mobilization, lymphocyte trafficking, cytokine tone, and endocrine suppression over time (14, 18, 19, 50, 52). Even if only a subset of tumors fully exploit tumor-brain circuits, the clinical implications could be substantial because systemic neuroendocrine signals reach every disease site.

Systems neurobiology becomes especially relevant when considering host state variables that are common in oncology but difficult to mechanistically integrate, such as chronic stress, depression, sleep disruption, pain, cachexia, and treatment-related physiologic strain. These conditions can alter sympathetic tone, hypothalamic-pituitary-adrenal output, and inflammatory set points, thereby influencing tumor immunity even when they do not directly change tumor genotype (14, 18, 19, 50). The neuro–immune framework offers a way to connect these host-level exposures with measurable tumor phenotypes.

## 3 Neuralized microenvironmental programs and metastatic adaptation

Metastatic dissemination requires more than migration. Cancer cells must survive transit, colonize a foreign tissue, and adapt to its trophic signals. This aligns with the broader concept that metastatic success depends on niche-specific adaptation, particularly in the brain, where a highly specialized microenvironment imposes distinct constraints on tumor survival and immune interaction (79). A growing body of work indicates that breast cancer accomplishes part of this adaptation by co-opting neural-developmental programs, including neurotrophin signaling, axon-guidance pathways, and adhesion programs that resemble neurovascular or axon-guided migration (20, 57, 85–90).

A compelling example is bone-to-meninges dissemination. Breast-cancer cells can migrate along emissary-vessel-associated routes and exploit NCAM-GDNF signaling in the meningeal niche, where macrophage-derived trophic factors support survival (57). This is not simply a metastasis story; it is a niche-construction story. Neural trophic logic is being repurposed as a survival system in a low-nutrient, immune-specialized environment.

Neurotrophin pathways are relevant beyond the meninges. NGF-TrkA signaling enhances migration, invasion, stemness, and chemoresistance in TNBC, while BDNF-TrkB signaling supports proliferation, endothelial interactions, recurrence risk, and brain-metastatic competence (20, 41, 42, 59). Although early NGF-receptor studies reported heterogeneous prognostic associations, the more consistent functional theme is that neurotrophin-responsive breast-cancer cells gain plasticity and resilience (20, 42).

Axon-guidance systems reinforce this adaptation. Netrin-1 suppresses dependence-receptor-mediated death and enhances metastatic survival; semaphorin-plexin signaling affects migration, lymphangiogenesis, immune composition, and distant dissemination (59, 60, 89, 91). In TNBC, direct migration along sensory fibers through PlexinB3-dependent mechanisms provides a particularly striking example of neuralized metastatic behavior (9, 60).

A further implication is that metastatic competence may not be a fixed intrinsic property. Exposure to neural cues at the primary site, in circulation-associated niches, or within distant organs may continually reprogram cancer cells toward more stem-like, stress-tolerant, and immune-evasive states. Longitudinal models that track neural exposure across metastatic progression are therefore needed, particularly for understanding brain, bone, and meningeal disease where local neural biology is unusually prominent (20, 39, 42, 57, 59, 60, 89, 91).

The metastatic route itself may also be neurally patterned. The recent demonstration that breast-cancer cells can exploit neural signaling pathways during bone-to-meninges dissemination provides a striking example of how anatomical transition zones rich in vascular and neural structures can become conduits for spread (57). Such routes are unlikely to be explained by tumor-cell motility alone. They imply that certain metastatic corridors may be preconditioned by neural architecture, inflammatory signaling, and immune permissiveness, creating combined highways of dissemination and immune tolerance.

Netrin and semaphorin pathways extend this logic. These molecules were classically studied in neural pathfinding, yet in cancer they can regulate survival, angiogenesis, immune-cell behavior, and stromal organization (59, 60, 89, 91). Their importance lies in their multifunctionality: they simultaneously affect how tumor cells move, how vessels and matrix are patterned, and how immune cells interpret the same tissue landscape. This makes them attractive conceptual bridges between metastasis biology and immune escape, even when the dominant effect in any given tumor remains context dependent.

RET/GDNF signaling illustrates this interface between developmental plasticity and therapeutic resistance. In breast cancer, RET activation has been associated with metastasis, endocrine resistance, and aggressive signaling states, while inflammatory cytokines can increase components of the pathway (20, 42). From an immunologic perspective, such pathways may do more than drive proliferation. By promoting survival, plasticity, and microenvironmental adaptability, they may reduce the effectiveness of immune-mediated elimination and facilitate the establishment of metastases that are inherently difficult to inflame.

These developmental-like neural programs are unlikely to depend solely on neuronal signaling itself and may also involve broader neural-supportive stromal interactions. In addition to neuronal signaling, nerve-supporting cells such as Schwann cells are increasingly recognized as active participants in metastatic niche remodeling (92). In response to neural injury or tumor-associated stress, Schwann cells can adopt repair-like phenotypes characterized by extracellular-matrix remodeling, cytokine secretion, and migratory support (93). Experimental studies further suggest that Schwann-cell-associated programs may facilitate tumor dissemination by generating permissive invasion tracks, promoting axon-guidance-like migratory behavior, and reshaping stromal interactions involving cancer-associated fibroblasts and immune cells (94, 95). Although their direct role in breast-cancer immune escape remains incompletely defined, these observations support the broader concept that neural regulation within tumors involves multicellular niche remodeling rather than neuronal signaling alone (96).

Metastatic adaptation can also be viewed as a form of developmental reprogramming in which cancer cells reacquire the ability to interpret neural and guidance cues that are normally used during tissue patterning. Increasing evidence suggests that this process may involve not only neuronal signaling itself but also broader neuralized microenvironmental remodeling, including interactions with Schwann cells and other stromal components (92, 94, 95). This is highly relevant to immune escape because migration, survival in transit, niche entry, and metastatic outgrowth all occur under immune pressure. Cells capable of decoding neurotrophins, axon-guidance ligands, or neural-metabolic signals may therefore be better equipped not only to colonize distant tissues but also to persist within partially immune-controlled environments (20, 42, 57, 59, 60, 89, 91).

## 4 Translational vulnerabilities and therapeutic opportunities

The translational question is whether neural biology can be exploited to improve immunotherapy or to restrain metastasis. The clearest near-term opportunity remains β-adrenergic blockade. Preclinical and early clinical data suggest that propranolol is feasible in breast cancer and may modulate biomarkers relevant to progression and immunity (24, 26, 30, 31, 80). ^A^ phase II trial is evaluating propranolol with pembrolizumab and chemotherapy in PD-L1-positive TNBC, reflecting a broader shift from purely tumor-cell-directed thinking toward immune-state modulation (82).

A second opportunity lies in the sensory-ECM axis. The recent breast-cancer-specific demonstration that sensory neurons can drive immune exclusion through CGRP-dependent matrix remodeling sharply increases the translational relevance of CGRP antagonism (29). This concept is further supported by recent work showing that collagen remodeling itself can act as an active driver of immune exclusion, for example through DDR1-dependent signaling that organizes dense collagen architectures and restricts T-cell infiltration (97). Because CGRP-targeting drugs already exist in neurology, repurposing logic is attractive. The key question will be patient selection: neural/ECM phenotyping is likely to matter more than histology alone.

Neurotrophin and receptor-tyrosine-kinase targeting represents a third axis. RET signaling is implicated in metastatic competence and therapeutic resistance, and selective RET inhibition has already shown activity in RET-altered solid tumors, including case-level evidence in breast cancer. TRK and related neurotrophin-receptor pathways may be relevant in selected receptor-positive or TNBC subsets, particularly where neural-trophic dependence is strong (20, 42, 59).

More speculative but potentially important interventions include dopamine-axis manipulation, targeted denervation, and strategies aimed at direct neuron-cancer signaling or TNT-mediated transfer (40, 51, 85). In parallel, emerging neuro–immune engineering strategies suggest that neural and glial cell populations may be actively reprogrammed to participate in immune responses. For example, recent work demonstrates that astrocytes can be engineered to express chimeric antigen receptors (CARs), enabling targeted recognition and clearance of pathological substrates (98). In addition, advances in immune cell trafficking indicate that anatomically specialized routes, such as skull–meningeal pathways, may be exploited to enhance immune cell delivery to neural niches (84). Although these approaches are primarily derived from non-oncologic contexts, they highlight a broader paradigm in which both immune cell routing and effector function can be precisely controlled at the neuro–immune interface. Nevertheless, they are valuable because they force the field to articulate what exactly should be targeted: nerve abundance, neuronal activity, mediator release, receptor usage, circuit reciprocity, or downstream stromal consequences. However, these concepts remain substantially less mature than β-blockade. For breast cancer specifically, the most realistic short-term translation is likely to come from phenotype-guided combination therapy rather than from monotherapy against a single neural mediator.

The perioperative period deserves special emphasis. Surgery, anesthesia, tissue injury, pain, and acute stress create a neuroimmune window in which catecholamine and glucocorticoid surges may affect tumor cell dissemination and immune competence. Although evidence remains incomplete, this window is biologically plausible for testing short-course neural modulation in conjunction with standard therapy. The potential clinical value is high because even modest improvements in control of residual disease could translate into meaningful long-term benefit (14, 18, 19, 23, 29, 81).

Neurotrophin and developmental-pathway targeting offers another route to translation. RET-directed therapy already has clear clinical precedent in other cancers, and case-level evidence suggests that RET-altered breast cancers can be therapeutically actionable (20, 80–83, 90). The immunologic opportunity may be greatest when these agents are used not merely as tumor-cell inhibitors but as components of rational combinations designed to disrupt niche adaptation, stromal signaling, and immune evasion simultaneously. Similar thinking may eventually apply to semaphorin-plexin or netrin-axis strategies if breast-cancer-specific biomarkers become sufficiently robust.

Sensory-axis intervention is currently less clinically mature than β-blockade but may ultimately prove just as important. CGRP antagonism, receptor blockade, nociceptor modulation, or TACR1-oriented strategies are attractive because they could act upstream of matrix hardening and neuropeptide-driven inflammatory suppression (12, 27–29, 36, 37). However, these approaches require caution. Sensory nerves also mediate normal tissue protection, repair, and pain signaling, so therapeutic benefit will likely depend on careful dosing, timing, and patient selection. Preclinical models must therefore evaluate anti-tumor efficacy alongside tissue homeostasis and treatment tolerance.

Combination design is likely to determine success. In adrenergic-dominant tumors, β-blockade may be best paired with checkpoint inhibition, myeloid-targeted agents, or therapies that restore antigen presentation. In sensory-exclusion-dominant tumors, neural targeting may need to be combined with matrix-modifying, vascular-normalizing, or CAF-directed strategies before checkpoint blockade can operate effectively. In interface-dominant metastatic disease, RTK inhibition or niche-directed therapy may be more rational than broad immune stimulation alone. The translational framework should therefore be phenotype based, not one-size-fits-all. A phenotype-guided therapeutic roadmap is presented in **Figure 4**.

The appeal of β-blockade lies partly in its availability, but its translational development should be more precise than simple repurposing. Not all β-blockers have identical receptor selectivity, pharmacokinetics, or central penetration, and not all patients are likely to derive equal benefit. The most plausible near-term scenario is biomarker-guided use in tumors with evidence of adrenergic activity, myeloid skewing, lymphovascular remodeling, or perioperative stress vulnerability rather than unselected administration to all patients with breast cancer (14, 15, 24–26, 30, 31, 80).

## 5 Biomarkers, technologies, and study design priorities

A major barrier to translation is the lack of standardized neural phenotyping in human breast tumors. Simple nerve-density measures are not sufficient. The field needs a layered biomarker framework that captures neural subtype, receptor state, spatial immune organization, stromal architecture, and systemic stress biology (5, 6, 16, 86–88, 99). Recent advances in single-cell and spatial transcriptomics, multiplex imaging, neural tracing, deep-tissue microscopy, tissue clearing, and proteomic mapping are beginning to enable higher-resolution characterization of neural–tumor ecosystems (34, 86–88, 99). At a minimum, future studies should distinguish adrenergic-high, sensory/ECM-dominant, and neural-interface/metastatic-adaptation phenotypes. These states are unlikely to have identical responses to β-blockade, CGRP inhibition, stromal therapy, or immunotherapy combinations. A simplified conceptual framework linking proposed neural phenotypes with candidate biomarkers and therapeutic strategies is summarized in **Table 1**.

**Table 1:** Conceptual neural phenotypes in breast cancer and their potential translational implications.

Neural phenotype	Dominant biologic features	Candidate biomarkers	Potential interventions	Representative references
Adrenergic-dominant	β-adrenergic signaling; myeloid suppression; stress-associated inflammation	ADRB2; TH+ fibers; MDSC/TAM enrichment	β-blockade (e.g., propranolol); stress-modulation strategies; immunotherapy combinations; perioperative sympathetic suppression	(14, 25, 26)
Sensory/ECM-dominant	CGRP signaling; CAF activation; collagen remodeling; immune exclusion	CGRP/RAMP1; COL1A1/COL3A1; myCAF signatures; CD8+ T-cell exclusion patterns	CGRP inhibition; stromal-targeting therapy; ECM remodeling approaches	(12, 27, 76)
Neural-interface/metastatic-adaptation	Synapse-like interfaces; TNT/organelle transfer; neural-metabolic coupling; niche adaptation	Glutamatergic markers; mitochondrial transfer; neural adhesion molecules; synapse-associated signaling	metabolic targeting; neural-interface disruption; anti-metastatic combinations; glutamatergic signaling inhibition	(40, 51, 75)
Systems neuroendocrine-dominant	CNS–tumor circuit activation; glucocorticoid signaling; chronic stress biology	cortisol signatures; inflammatory markers; neuroendocrine profiles; glucocorticoid-response programs;	behavioral interventions; perioperative modulation; β-blockade; neuroendocrine-targeted combinations; circadian/sleep regulation strategies	(19, 49, 52)

Experimental design should also move beyond static endpoint measurements. Neural influences are dynamic and state-dependent. A pathway that enhances acute inflammation may ultimately generate chronic exclusion or metastatic selection. Longitudinal sampling before, during, and after therapy is therefore essential if neural biomarkers are to become clinically useful.

Finally, future studies should explicitly incorporate clinical heterogeneity. Subtype, menopausal status, obesity, pain burden, psychosocial stress, circadian disruption, treatment history, and metastatic organotropism may all influence neural biology. These variables are often treated as confounders, but they may in fact be part of the causal architecture. A mature breast-cancer neuroimmunology field will need prospective biobanking and metadata collection broad enough to model these interactions rather than average them away.

Causality requires intervention-based study design. Correlating nerve signatures with immune phenotypes is useful, but it does not establish whether nerves are upstream drivers, parallel markers, or downstream consequences of aggressive biology. Stronger studies will manipulate neuronal activity, specific neuropeptide pathways, receptor usage, or circuit access while simultaneously measuring immune topology and treatment response. The most convincing evidence will come from experiments in which neural intervention converts a resistant immune phenotype into a permissive one.

Model choice is equally critical. Many current systems emphasize rapid tumor growth but inadequately capture chronic stress, persistent pain, stromal maturation, or metastatic dormancy. Preclinical designs should incorporate longitudinal neural manipulation, behavioral and endocrine readouts, and clinically relevant treatment schedules, including neoadjuvant and perioperative contexts (18, 19, 50, 52, 53, 55, 58). Human ex vivo platforms, organoids with neural or stromal components, and co-culture systems with immune cells will also be needed to bridge mechanistic precision with translational relevance.

Spatially resolved technologies will be central not simply for mapping nerves, but for determining how neural signals organize immune and stromal architecture within tumors. Single-cell RNA sequencing can identify receptor-ligand opportunities, but spatial transcriptomics and multiplex imaging are needed to determine whether immune cells are trapped at sensory-rich stromal fronts, whether TAMs accumulate along adrenergic tracks, or whether metastatic lesions form interface-competent neural niches (18, 50, 52, 53, 57, 77). Without spatial data, neural programs may appear diluted or misleading in bulk assays, particularly when only small subregions of the tumor are neurally dominant.

Biomarker development should begin with pathology but not end there. Standardized quantification of nerve density, nerve subtype, perineural proximity, and neurotrophin expression can provide an accessible first layer, especially if integrated with immune-cell topology and stromal architecture on the same section. However, crude nerve counts alone are unlikely to be sufficient. Functional state matters: a sparsely innervated but highly active neural niche may be more immunologically consequential than a densely innervated but quiescent one. This argues for multimodal biomarkers that combine morphology with transcriptional and protein-level indicators of signaling activity (8–10, 15, 16, 27–29, 50).

## 6 Discussion and conclusion

The main contribution of the neuro–immune framework is conceptual. It explains why immune failure in breast cancer is not reducible to checkpoint-ligand expression or to broad labels such as ‘hot’ and ‘cold’. Neural systems influence whether immune cells remain functional, whether effective immune penetration into the tumor core is achieved, and whether disseminated tumor cells successfully adapt to specialized tissues (4, 5, 7, 27, 40).

Importantly, not all mechanisms reviewed here are equally established in breast cancer. The strongest breast-cancer-specific evidence currently supports sympathetic β-adrenergic regulation, sensory-nerve-associated structural remodeling, PlexinB3-linked nerve-guided migration, neurotrophin-dependent metastatic adaptation, and circuit-level links between the brain and tumor progression (5–7, 29, 36, 57, 58). By contrast, several mechanisms discussed in this review remain more strongly supported by evidence from glioma, pancreatic cancer, or other neurotropic malignancies than by direct breast-cancer-specific validation. These include true synaptic integration and electrophysiologic neuron–tumor coupling observed in glioma models, TNT-mediated nerve-to-cancer organelle transfer during metastasis, and portions of the neuroinflammation literature derived primarily from brain-tumor and broader cancer-neuroscience studies (5, 51, 75, 77, 100). Accordingly, some concepts discussed here should currently be viewed as biologically informative frameworks rather than definitively established breast-cancer mechanisms.

An important challenge in cancer neuroscience is that neural regulation does not appear to function as a uniform oncogenic program across all tumor contexts. Several mechanisms discussed in this review show substantial variability depending on tumor type, disease stage, anatomical location, and experimental model (101). For example, sensory-neuron-associated signaling has been linked to stromal remodeling and immune exclusion in breast cancer and other solid tumors, whereas neuronal activity in different contexts may also transiently support inflammatory recruitment or tissue-repair programs (12, 27). Similarly, highly organized synapse-like neuron–tumor interfaces are strongly supported in glioma and certain neurotropic malignancies, while breast cancer more commonly demonstrates neural adaptation, nerve-guided migration, and metabolic coupling rather than fully established electrophysiologic synaptic circuitry (9, 39–41, 57, 75, 77, 100). These discrepancies likely reflect genuine biologic context dependency rather than simple inconsistency. Acute, transient neural activity should also be distinguished from chronic adrenergic or neuroendocrine conditioning, because prolonged stress-associated catecholamine signaling can progressively remodel immune, stromal, and lymphovascular compartments, while glucocorticoid signaling may further suppress antigen presentation, NK-cell activity, and cytotoxic immune responses (14, 15, 18, 19, 32, 49).

This context dependency is particularly important within breast cancer itself. Breast-cancer models differ substantially in subtype representation, implantation route, tumor-cell dose, growth kinetics, metastatic capacity, stromal maturation, and immune contexture. Comparative studies of intraductal TNBC models have shown that 4T1- and Py230-based tumors exhibit distinct progression patterns and immune microenvironments, with aggressive 4T1 tumors showing stronger inflammatory and metastatic features, whereas Py230 tumors display a more immunosuppressed profile (102). More recent comparative analyses of multiple syngeneic TNBC models further demonstrate marked heterogeneity in interferon signaling, metabolic programs, growth kinetics, metastatic capacity, splenic immune reactions, and adaptation to PD-L1 blockade (103). These observations are highly relevant for cancer neuroscience because the same variables that shape tumor immunity are also likely to influence neurotrophic signaling, nerve recruitment, and the downstream consequences of neural input.

Tumor burden is another critical variable. In the 4T1 model, the number of inoculated cells can strongly influence growth dynamics and metastatic efficiency, and large inocula may generate rapidly expanding tumors that incompletely recapitulate the slower stromal and immune remodeling of human breast cancer (104). Implantation site also matters: orthotopic and subcutaneous 4T1 models differ in tumor growth, vascular density, lung metastasis, and histopathologic features, suggesting that neural and immune architecture should be interpreted in relation to local tissue context (105). Finally, host immune status is essential when evaluating neuro–immune mechanisms. Immune-competent syngeneic or autochthonous models are better suited for studying neural regulation of myeloid cells, T cells, NK cells, dendritic-cell priming, and immune exclusion, whereas immune-deficient xenografts may be more informative for tumor-intrinsic neural adaptation but less suitable for assessing immune escape (106). Future breast-cancer neuroscience studies should therefore report tumor model, implantation route, tumor-cell number, tumor size at analysis, host immune competence, immune-cell composition, stromal maturity, and nerve-detection methods in sufficient detail to support cross-study interpretation.

Taken together, the literature supports a model in which nerves operate across four linked levels: functional immune programming, spatial gatekeeping, direct signaling interfaces, and systemic state control. This multiscale view has immediate translational implications. Beyond this multiscale framework, the temporal dynamics of neural signaling may also carry important therapeutic implications, as perioperative and neoadjuvant windows could provide opportunities for transient neural-targeted interventions aimed at limiting acute stress-associated immune suppression and metastatic conditioning. Rather than treating all immunotherapy-resistant breast cancers as biologically similar, a neural-state taxonomy may identify subgroups that are more likely to benefit from β-blockade, CGRP-axis intervention, stromal decompression, or brain-penetrant targeted therapy (34). Neural biology should therefore be treated not as an optional annex to tumor immunology, but as part of the core explanatory framework for immune escape in breast cancer.

Current evidence supports a coherent, testable model in which nerves help determine whether breast cancer remains immunologically visible, spatiotemporally regulated, and systemically controllable. Sympathetic signaling programs suppressive immune states; sensory signaling organizes exclusionary tissue geometry; direct neuron-cancer interfaces enhance fitness and metastatic resilience; and central circuits regulate the host background against which all of these processes unfold (7, 12, 20, 27–29, 36–42, 51–53, 57, 59, 60, 89, 91). As experimental tools and clinical datasets improve, neuro–immune phenotyping may become an integral part of precision immuno-oncology in breast cancer rather than a peripheral curiosity.

Equally important is restraint. The field should avoid replacing one reductive narrative with another. Neural regulation is unlikely to be the dominant determinant of every breast-cancer immune phenotype, and some striking mechanisms discovered in pancreatic cancer, glioma, or head and neck cancer may prove to be only partially relevant in mammary tumors (27, 40, 75). Indeed, recent studies suggest that neural regulation may exert bidirectional and context-dependent effects across tumor types and microenvironmental states. While adrenergic and neurotrophin signaling frequently drive tumor growth and stromal remodeling in pancreatic cancer models, cholinergic signaling has been reported to exert partially opposing effects in certain contexts (17, 107). Similarly, sensory or sympathetic neuronal activity may restrain tumor progression under specific immune conditions (108, 109). For example, sympathetic axons have been shown to restrain melanoma growth through α2-adrenergic signaling and modulation of pro-tumor myeloid-cell distribution, highlighting that neural influences on tumor immunity cannot be universally classified as tumor-promoting (78). The value of the neuro–immune model lies not in universal applicability but in its capacity to explain a subset of clinically important resistance states that are otherwise difficult to categorize (110).

In conclusion, the broader significance of this framework is that it reframes immune escape as an emergent property of cross-system integration. Genomic alterations, stromal remodeling, developmental plasticity, and host physiology do not operate in isolation; nerves provide one of the few biologic infrastructures capable of linking them across scales. This is why the field has moved so quickly from descriptive observations of tumor innervation to intervention-oriented questions about whether neural circuits can be therapeutically rewired (5–7, 16).

## 7 Future directions for neuro-immunology-guided breast cancer research

A decisive next step for the field is to move from descriptive co-localization to intervention-guided immunobiology. Many current datasets can tell us that neural markers coexist with specific immune phenotypes, but far fewer can show which neural signals are necessary for maintaining those states, when during disease evolution they matter most, and whether interrupting them produces durable changes in anti-tumor immunity. Future studies should therefore prioritize designs in which neural pathways are manipulated before, during, and after standard therapy while immune topology, metastatic burden, and host neuroendocrine state are measured in parallel (12, 14, 18, 19, 23, 27–29, 50, 52, 53, 55, 58). Such designs are more demanding, but they are essential if breast-cancer neuroimmunology is to progress from a compelling narrative to a clinically useful discipline.

A second priority is subtype-aware and organ-aware stratification. The field should resist the temptation to treat breast cancer as a single neural ecosystem. TNBC is currently the most informative entry point because it offers higher immunogenicity and clearer evidence of neural remodeling, yet luminal disease, endocrine-resistant metastasis, brain metastasis, and meningeal dissemination may rely on different combinations of sympathetic, sensory, developmental, and metabolic neural cues (20, 39, 42, 57, 59, 60, 89, 91). Likewise, primary tumors, lymph-node metastases, bone lesions, brain metastases, and meningeal disease should not be expected to share the same neural logic. A rigorous translational program will therefore need matched sampling across sites and disease stages, with explicit modeling of how neural state changes during treatment and dissemination.

Third, the field needs trial concepts that are explicitly neuro-immunologic rather than merely drug-repurposing exercises. For example, perioperative β-blockade should be tested with immune, stromal, and circulating-tumor-cell endpoints that reflect its hypothesized mechanism, not only with generic pathologic response measures. Sensory-axis interventions should be evaluated in tumors with pre-existing evidence of exclusionary matrix architecture, nociceptor signatures, or neuropeptide activity rather than in unselected populations. RTK- or developmental-pathway-targeted combinations should be built around niche adaptation hypotheses in metastatic settings where those pathways are biologically active (20, 80, 82, 83, 90). In each case, the principle is the same: align trial eligibility, pharmacodynamic readouts, and clinical endpoints with the specific neural failure mode being targeted.

Finally, breast-cancer neuroimmunology will likely benefit from closer integration with adjacent disciplines that have historically been studied separately: psychoneuroimmunology, pain biology, metastasis research, stromal ecology, and spatial omics. The most informative future models may be those that combine patient-reported stress or pain measures, endocrine biomarkers, advanced imaging, multiplex spatial pathology, and longitudinal biospecimen collection. Such integrative programs are difficult, but they are uniquely positioned to answer the questions that matter most for patients: which tumors are neurally organized, when neural biology becomes actionable, and whether intervening on neuro–immune crosstalk can meaningfully improve the depth and durability of anti-cancer immunity. If these questions can be answered with the same rigor now being applied to genomic precision oncology, neural biology may become a genuine axis of precision immunotherapy in breast cancer rather than an ancillary explanatory layer. In parallel, consensus reporting standards for neural markers, immune topology, and neuroendocrine metadata would substantially improve cross-cohort comparability and accelerate meta-analytic validation.

### 7.1 Toward clinically actionable neuro–immune phenotypes

A practical next step for the field is to define neuro–immune phenotypes that are useful not only descriptively but also operationally. At present, most studies classify breast tumors by receptor subtype, immune abundance, or metastatic site. Those variables remain essential, yet they do not fully capture whether a given tumor is neurally organized. A more actionable framework would stratify tumors according to the dominant mode by which neural signals sustain immune failure. One phenotype would be adrenergic-dominant, characterized by high β-adrenergic receptor expression, stress-linked transcriptional programs, myeloid skewing, reduced dendritic-cell competence, and evidence of lymphatic or vascular remodeling (14, 15, 21–26, 30, 31). ^A^ second would be sensory-exclusion-dominant, defined by nociceptor enrichment, CGRP-axis activity, fibroblast activation, collagen-dense extracellular matrix, and marked CD8 sequestration at the invasive margin rather than within the tumor core (12, 27, 29). ^A^ third would be interface-dominant, in which direct neuron-cancer communication, neurotransmitter utilization, pseudo-synaptic signaling, or mitochondrial transfer appears to confer metabolic and survival advantages that are not fully explained by classic checkpoint biology (38–41, 51). ^A^ fourth phenotype would be systems-neuroendocrine-dominant, where chronic stress, circadian disruption, or tumor-brain-sympathetic reciprocity lowers the host immune set-point and amplifies otherwise modest local suppressive programs (18, 19, 50, 52, 53, 55, 58).

These phenotypes should not be treated as mutually exclusive boxes. Many aggressive tumors are likely to combine features from more than one class. Nevertheless, this framework is useful because it links pathology, mechanism, and intervention. For example, a tumor with high β2-AR activity, abundant CD11b+ suppressive myeloid cells, and strong stress-hormone signatures may be better suited to β-blockade-based combination strategies than a tumor whose dominant feature is dense collagen and severe spatial exclusion (24–26, 30, 31, 80). Conversely, a lesion enriched for sensory fibers, CGRP-associated fibroblast programs, and edge-restricted T cells may be more rationally approached through matrix-disrupting or sensory-axis-directed therapy than through escalation of checkpoint inhibition alone (12, 27, 29). Likewise, interface-dominant tumors may require attention to metabolic or neurotransmitter dependency, particularly in brain-tropic or highly plastic metastatic states (39–41, 51).

The immediate translational value of such phenotyping lies in clinical-trial enrichment. Many negative immunotherapy combinations in breast cancer likely fail not because the biological idea is wrong, but because the enrolled population contains multiple immune-failure mechanisms that are not equally targetable. Neural phenotyping could therefore help distinguish tumors that are immunologically silent because they cannot be primed from those that are actively excluded, chronically stress-amplified, or metabolically rescued by neural support. From a biomarker-development standpoint, this argues for layered assessment rather than single-marker selection. Histologic nerve density alone is unlikely to be sufficient. More informative composite panels would combine nerve-subtype markers, receptor state, spatial immune architecture, stromal compression or collagen signatures, and selected systemic indicators such as catecholamine exposure or glucocorticoid-linked immune suppression (14–16, 18, 19). If validated prospectively, these phenotypes could move neuro-immunology from an explanatory framework to a patient-selection tool.

### 7.2 Integrating neural variables into immunotherapy study design

A related priority is to determine how neural biology should be incorporated into the design of breast-cancer immunotherapy studies. The most obvious opportunity is combination therapy, but mechanism must come before empiricism. In adrenergic-high disease, β-blockade is unlikely to function as a conventional cytotoxic partner; its value would be to reduce myeloid suppression, improve effector-cell fitness, and blunt dissemination-associated stress signaling during periods when anti-tumor immunity is being mobilized (24–26, 30, 31, 80). That makes timing crucial. Short perioperative courses, neoadjuvant windows, or early-treatment combinations may be biologically more appropriate than salvage-stage monotherapy. By contrast, sensory-exclusion-dominant tumors may derive limited benefit from immune activation unless the physical barrier to T-cell entry is also addressed. In such settings, the relevant end points may include not only response rate but also spatial redistribution of CD8-positive cells, stromal decompression, collagen organization, and conversion from edge-restricted to core-penetrant immunity (12, 27, 29).

Neural variables should also influence sample collection. If the central question is whether an intervention reprograms a neurally maintained immune state, then pretreatment and on-treatment tissue should be analyzed with spatial methods capable of preserving nerve-immune-stroma relationships. Multiplex immunofluorescence, digital pathology, spatial transcriptomics, and targeted proteomics are particularly suitable because they can localize receptor expression and quantify whether immune cells move from the margin into the core after treatment (16, 29, 60). Blood-based biomarkers may still be useful, especially for stress and neuroendocrine readouts, but they should be interpreted as complementary rather than definitive. Circulating catecholamines or inflammatory mediators can support biological interpretation, yet they cannot replace direct assessment of tumor architecture and local cellular state.

Finally, trial design should account for disease stage and organ context. Neural dependencies in minimal residual disease, loco-regional recurrence, and established visceral metastasis are unlikely to be identical. The perioperative window may be optimal for testing stress-axis modulation because surgery and tissue injury create a transient but biologically intense neuroimmune perturbation (14, 18, 19, 23, 32, 80). In contrast, metastatic trials may require organ-specific enrichment. Brain-tropic or leptomeningeal disease could be especially informative for studying interface-dependent or neurotrophin-dependent mechanisms, whereas lung-tropic disease may better reveal stress-conditioned niche biology and neutrophil-NETosis interactions (39, 50, 57). This stage-aware framework is particularly important because it reframes immunotherapy resistance as a spatially and temporally structured problem. The central question is not simply whether neural biology matters, but when it matters most, in which immune context, and at which stage of disease progression intervention is most likely to alter the disease trajectory.
